# 

**Published:** 1881-04

**Authors:** 


### THE BISTOURY:

Thad S. Up de Graff, M. D., Editor & Proprietor.

### *CONTENTS FOR APRIL, 1881.*

**CONTRIBUTIONS.** PAGE.

Capital Impressions ........................... BEN. H. PRATT ........................... 1
The Book Wonderful, ........................... AUSBURN TOWNER ........................... 4
Protoplasm ........................... S. O. GLEASON, M. D. ........................... 6
Words and Slang ........................... J. B. CHANDLER ........................... 7
To Prevent the Spreading of Scarlet Fever.. J. DAVIDSON, M. D. ........................... 8

**MEDICAL ITEMS AND NEWS.**

Syphilitic Neuralgia—Styptic Collodion—Cystytis—Diphtheria—Esmarck's Caustic Powder—Burns—Remedies for Chilblains—Syphilis—Uterine Engorgement—Urethritis—Battey Solution for Eczema—Tonic Glycerine—Soda Mint—Gleet—Dropsy—Barber's Itch—Fever and Ague—Abdominal Typhus by the Douche—Herpes Circinati, Herpes Zoster, and Herpes of the Prepuce—Leucorrhœa, or Vaginal Catarrh—Gonorrhœa and Vaginits—A Menstruum for Salicylic Acid—Castor Oil—Microscopic Examination of Blood—Argyria Following the Frequent Pharyngeal Applicaton of Nitrate of Silver—Vapors for Inhalation—Injections of Tincture of Iron in Nœvus—Cheken—Chest Affections—Tonga—Chlorate of Potassium and the Increase of Nephritis—Purgatives in Phthisis Diarrhœa of Infants—Itch—Balsam of Peru in Pruritus—Vermifuge—Cystitis—Asthma—Compound Camphor Powder—Salicylic Acid for Bee Stings—Nitro-Glycerine—Pharyngitis—Heart Disease—Formula for Hypodermic Use in Syphilis—Small Pox Pitting—Whooping Cough—Strychnia as a Physiological Antidote to Alcohol—Diphtheria—Chronic Bronchitis—Iodide of Potassium in Cardiac Dyspnœa ........................... 10 17

**OUR CORRESPONDENCE** Six Letters with answers ........................... 18

**HOUSEHOLD HINTS AND RECIPES.**

That Potato Salad—Furniture Polish—To fix Pencil Marks—Rust—A Good Elastic Glue—Cement for Leather—Transparent Paint for Glass—Distinguishing the Diamond—Diamond Ink—Drilling Glass—Tallow for the Carpet-Beetle—The Preservation of the Teeth ........................... 19 20

**EDITORIAL DEPARTMENT.**

New Type—Ben. H. Pratt in Washington—Premium List—Bodines—Change in Editorial Headings—Error in Title Page—The New President—Our Sunday Papers—Elmira College—Schwab—General Hancock—Surgeon General—Specimen Copies—A New York Cremation Society—A Foot Bridge—Generosity—Dr. A. J. Ingersoll—"Do We Owe Our Parents Anything?"—Clean Up—Coroner's Inquests—Kissing—Microscopy—A Cat-astrophy—Sara's Art Gallery—Another Edison Humbug ........................... 22 26

